# Phylogeography of the Korean endemic *Coreoleuciscus* (Cypriniformes: Gobionidae): the genetic evidence of colonization through Eurasian continent to the Korean Peninsula during Late Plio-Pleistocene

**DOI:** 10.1007/s13258-022-01243-y

**Published:** 2022-04-19

**Authors:** Hyung-Bae Jeon, Ha Youn Song, Ho Young Suk, In-Chul Bang

**Affiliations:** 1 Department of Biology, Concordia University, Montreal, Canada; 2 Inland Fisheries Research Institute, National Institute of Fisheries Science, Busan, South Korea; 3 Department of Life Sciences, Yeungnam University, Gyeongsan, South Korea; 4 Department of Life Sciences, Soonchunhyang University, Asan, South Korea

**Keywords:** Phylogeography, *Coreoleuciscus*, Endemism, mtDNA, COI

## Abstract

**Background:**

Freshwater endemism is thought to have been formed through the vicariance of connected water systems or the process by which ancestral populations colonized specific areas. The Korean Peninsula is well recognized for its high level of freshwater endemism with about 40% of freshwater fish species being endemic.

**Objective:**

In this study, we attempted to reconstruct the process of speciation and phylogenetic dispersal of *Coreoleuciscus* species, which is endemic in the Korean Peninsula.

**Methods:**

We used fossil-calibrated divergence time estimation and ancestral distributional reconstruction to infer phylogeographic reconstruction of *Coreoleuciscus* based on mitochondrial cytochrome c oxidate subunit I (COI) sequences (1551 bp).

**Results:**

Our phylogeographic analysis based on a total of 626 individuals revealed that the two *Coreoleuciscus* species have originated from the independent colonization of different lineages in the ancestral populations, probably during the Late Plio-Pleistocene. The full-scale expansion of *Coreoleuciscus* populations appears to have taken place after major river structures were completed on the Korean Peninsula. We also provided evidence that the common ancestors of *Coreoleuciscus* was distributed in Eastern Eurasian continent and subsequently dispersed into the tip of East Asia. High genetic diversity was mainly concentrated in large drainage populations, while small populations showed an monomorphism, which could give important implications for planning the conservation and management of *Coreoleuciscus*.

**Conclusions:**

The phylogenetic background of the rheophilic *Coreoleuciscus* species can be explained by the colonizer hypothesis that the endemic freshwater fish originated from the common ancestor in continental region.

**Supplementary Information:**

The online version contains supplementary material available at 10.1007/s13258-022-01243-y.

## Introduction

Freshwater endemism is thought to have been formed through the vicariance of connected water systems or a process by which ancestral populations colonized into the new areas (Reyjol et al. 2007; Tedesco et al. 2012). The Korean Peninsula is well recognized for its high level of freshwater endemism with about 40% of freshwater fish species being endemic (Choi 1973; Yoon et al. 2018). This peninsula can be divided into three major biogeographic subdistricts based on ichthyofauna; west Korea subdistrict (WKS), south Korea subdistrict (SKS) and northeast Korea subdistrict (NKS) (Kim 1997). Several hypotheses have been proposed to explain what the historical driver was for such a high level of endemism appearing in such a small area. The ‘Speciation by mountains’ hypothesis explains that uplifts of mountains probably have frequently prevented gene flow among populations, leading to allopatric speciation (Choi 1973; Kim and Bang 2012; Kim et al. 2012; Kwan et al. 2017). This hypothesis is supported by the results of studies of Korean cobitid species, which are presumed to have speciated during the Miocene (Kim and Bang 2012; Kwan et al. 2017), and *Coreoleuciscus* species (Kim et al. 2012).

In contrast, the ‘colonizer hypothesis’ explains that frequent colonization of independent lineages from the Eurasian continental ancestral population may be the major cause of high endemism (Bilton et al. 1998; Reyjol et al. 2007). Molecular phylogeographic data from previous studies have revealed that the speciation and intraspecific population structure of some Korean bitterlings may have arisen from dispersal after colonization of continental ancestors during the Pleistocene and subsequent vicariance between WKS and SKS drainages (Jeon 2018; Won et al. 2020). The rivers flowing to the southwest coast of the Korean Peninsula formed a confluence with the present Yellow Sea, the paleo-Yellow River, during the Pleistocene, when the sea levels were lower than at present (Zhang et al. 2019). Since then, sea levels have risen, and the populations living in the freshwater system of this region have the isolated distribution as they are now. The colonizer hypothesis is supported as more plausible, at least partially, considering the paleo-geographic context of this area where about 70% of Korean species occur.

*Coreoleuciscus* (Cypriniformes: Gobionidae) is a small freshwater fish endemic to the Korean Peninsula (Kim 1997; Song and Bang 2015). This genus has long been considered as monotypic until *C. aeruginos* was reported as a new species (Song and Bang 2015). The allopatric distributional range of the two *Coreoleuciscus* species is bisected by mountainous terrain; *C. aeruginos* in SKS and *C. splendidus* in WKS (Song et al. 2010), which provides an opportunity to test the two phylogeographic hypotheses for freshwater species diversity by reconstructing the spatiotemporal pattern of dispersal and divergence. No study has yet been conducted to examine the phylogeographic patterns of these two species with extensive geographic coverage. A previous molecular phylogenetic study showed that these two *Coreoleuciscus* species diverged at 37.9 MYA (26.37–51.94, 95% CI), the Oligocene-Eocene, when the Baekdudaegan Mountain Range (BMR) was formed, which caused spatial separation between subdistricts of the Korean freshwater system (Kim et al. 2012). However, it should be noted that attempts at molecular dating based on substitution rates of mtDNA tend to result in overestimation of divergence time (Kim and Bang 2012; Kim et al. 2012; Zhao et al. 2016). Indeed, there are numerous cases in which the divergence time of the major nodes in the empirically obtained phylogenetic trees differed greatly from the fossil-calibrated divergence time due to such overestimation (Near et al. 2012; Zhao et al. 2016; Kim et al. 2017).

In this study, we analyzed mitochondrial cytochrome c oxidate subunit I (COI) sequences obtained from 626 *Coreoleuciscus* individuals that were collected throughout the entire range on the Korean Peninsula. Two major tools were attempted to reconstruct the phylogeographic history of *Coreoleuciscus* based on this genetic information; fossil-calibrated Bayesian inference using BEAST to determine divergence times, and Bayesian Binary MCMC (BBM) analysis to determine the ancestral ranges of major phylogenetic clades. Through these analyses, we expected to confirm whether the speciation of *Coreoleuciscus* occurred with post-dispersion isolation of ancestral populations that colonized during the Pleistocene, similar to the pathway suggested in the studies of bitterlings (Jeon 2018; Won et al. 2020). More specifically, the colonizer hypothesis can be supported if the phylogenetic analysis showed that ancestral lineage of both species was distributed in out of the Korean Peninsula after the formation of the mountains. On the contrary, if the speciation of the two *Coreoleuciscus* species has occurred when the BMR was formed, then the speciation by mountains hypothesis can be supported. The results of this study are also expected to provide an opportunity to construct a reliable DNA barcode library for *Coreoleuciscus* species covering the entire distributional range.

## Materials and methods

### Sample and DNA extraction

**Fig. 1:**
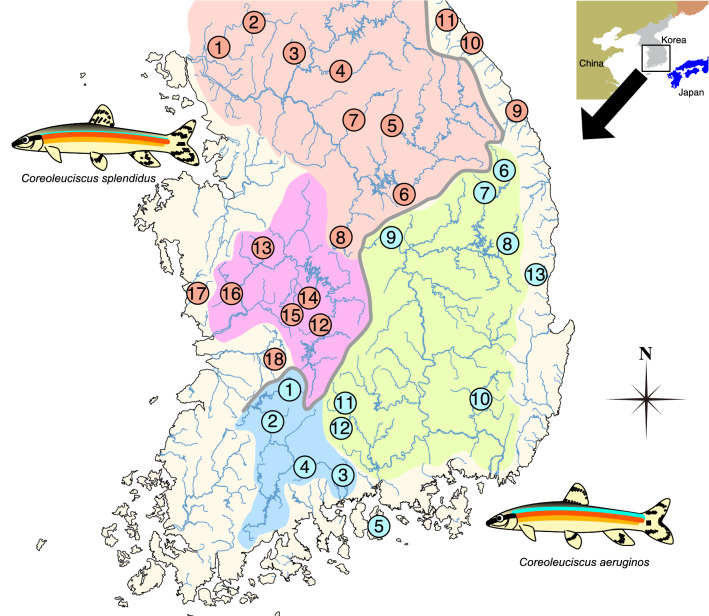
Sampling sites and distribution area of *Coreoleuciscus* species on the Korean Peninsula; sheds represent the major river basins (red: Han River, pink: Geum River, blue: Seomjin River, Green: Nakdong River). The grey line indicates the Baekdudaegan Mountain Range (BMR). The number in circle indicates the population code. The red and blue circles indicate *C. splendidus* and *C. aeruginos*, respectively (color figure online)

A total of 626 *C. splendidus* and *C. aeruginos* individuals were collected from 31 locations in South Korea (Fig. 1) from 2007 to 2015. Sixteen to twenty-four individuals were collected from each location (Table 1). The voucher specimens preserved in 80% ethanol were deposited in the collection for Department of Life Science and Biotechnology at Soonchunhyang University in South Korea. Genomic DNA (gDNA) was extracted from a left pelvic fin using the conventional SDS/proteinase K method (Sambrook and Russell 2012). The gDNA extracted was resuspended in 1 × TE buffer (10 mM Tris-HCl; 1 mM EDTA, pH 8.0), and the concentration and quality were checked using a NanoDrop 1000 (Thermo Fisher Scientific, Wilmington, DE, USA).

**Table 1:** Sampling sites and vouchers of *Coreoleuciscus* species analyzed in this study

Species	River	Drainage	Code	Map code	N	GPS coordinate	Voucher no.
CS	Imjin River	Han	CS-H-IJ	1	20	38° 05′ 31.98″ N 127° 05′ 31.98″ E	SUC1338-1357
CS	Hantan River	Han	CS-H-HT	2	20	38° 09′ 35.44″ N 127° 24′ 37.64″ E	SUC2420-2439
CS	Jojong stream	Han	CS-H-JJ	3	20	37° 44′ 20.00″ N 127° 26′ 04.63″ E	SUC1313-1332
CS	Hongcheon River	Han	CS-H-HC	4	20	37° 41′ 59.02″ N 127° 41′ 49.04″ E	SUC2231-2250
CS	Pyeongchang River	Han	CS-H-PC	5	20	37° 25′ 34.78″ N 128° 23′ 09.73″ E	SUC2124-2143
CS	Hail stream	Han	CS-H-HI	6	18	37° 00′ 46.29″ N 128° 23′ 56.85″ E	SUC2489-2506
CS	Seom River	Han	CS-H-SG	7	20	37° 18′ 14.94″ N 127° 48′ 27.52″ E	SUC2163-2182
CS	Dal stream	Han	CS-H-DC	8	20	36° 39′ 22.52″ N 127° 44′ 23.38″ E	SUC2256-2275
CS	Samcheokosip stream	Samcheokosip	CS-H-SO	9	20	37° 24′ 25.38″ N 129° 06′ 41.44″ E	SUC2035-2054
CS	Gangneungnamdae stream	Gangneungnamdae	CS-H-GN	10	16	37° 43′ 15.05″ N 128° 49′ 00.44″ E	SUC2148-2162
CS	Yeongok stream	Yeongok	CS-H-YG	11	20	37° 51′ 00.14″ N 128° 45′ 36.69″ E	SUC4689-4708
CS	Upper Geum River	Geum	CS-G-SD	12	20	36° 08′ 05.24″ N 127° 39′ 30.19″ E	SUC1383-1402
CS	Yugu stream	Geum	CS-G-YG	13	20	36° 28′ 39.69″ N 127° 02′ 47.33″ E	SUC2076-2095
CS	Yudeung stream	Geum	CS-G-YD	14	23	36° 09′ 33.33″ N 127° 23′ 48.22″ E	SUC8862-8884
CS	Gap stream	Geum	CS-G-GC	15	22	36° 11′ 48.50″ N 127° 17′ 33.78″ E	SUC8832-8853
CS	Goemokdong stream	Geum	CS-G-GM	16	20	36° 05′ 28.78″ N 127° 16′ 39.04″ E	SUC2467-2486
CS	Ungcheon stream	Ungcheon	CS-U-UC	17	20	36° 18′ 51.43″ N 126° 39′ 25.48″ E	SUC2377-2396
CS	Soyang stream	Mangyeong	CS-M-SY	18	20	35° 50′ 50.07″ N 127° 15′ 53.28″ E	SUC2352-2371
CA	Jowon stream	Seomjin	CA-S-JW	1	20	35° 41′ 21.89″ N 127° 16′ 52.69″ E	SUC1360-1379
CA	Middle Seomjin River	Seomjin	CA-S-SC	2	24	35° 23′ 34.17″ N 127° 12′ 33.13″ E	SUC8952-8975
CA	Hwagae stream	Seomjin	CA-S-HG	3	20	36° 28′ 40.73″ N 127° 02′ 48.91″ E	SUC2098-2117
CA	Gurim stream	Seomjin	CA-S-GR	4	20	35° 27′ 34.06″ N 127° 06′ 25.69″ E	SUC2328-2347
CA	Samhwa stream	Samhwa	CA-S-SH	5	19	34° 48′ 19.71″ N 128° 01′ 57.43″ E	SUC2057-2075
CA	Upper Nakdong River	Nakdong	CA-N-BH	6	20	36° 55′ 52.62″ N 129° 00′ 19.80″ E	SUC2278-2297
CA	Hwangji stream	Nakdong	CA-N-HJ	7	20	37° 05′ 22.32″ N 129° 02′ 40.29″ E	SUC7841-7861
CA	Banbyeon stream	Nakdong	CA-N-BB	8	20	36° 42′ 03.46″ N 129° 07′ 57.20″ E	SUC9021-9040
CA	Yeong River	Nakdong	CA-N-YG	9	20	36° 39′ 46.44″ N 128° 01′ 19.87″ E	SUC2304-2323
CA	Milyang River	Nakdong	CA-N-MY	10	20	35° 33′ 07.25″ N 128° 45′ 33.36″ E	SUC2397-2416
CA	Gyeongho River	Nakdong	CA-N-GH	11	20	35° 27′ 48.95″ N 127° 47′ 22.50″ E	SUC2442-2461
CA	Deokcheon River	Nakdong	CA-N-DC	12	20	35° 16′ 29.06″ N 127° 50′ 33.69″ E	SUC1290-1309
CA	Yeongdeokosip stream	Yeongdeokosip	CA-N-YO	13	24	36° 23′ 17.36″ N 129° 18′ 21.51″ E	SUC8989-9013

CS (red circles in Fig. 1) and CA (blue circles in Fig. 1) in the species column stand for the *C. splendidus* and *C. aeruginos*, respectively

### PCR and sequencing

The Cytochrome c oxidase subunit I (COI) gene was amplified in a 20 μl reaction volume including AccuPower PCR Premix (Bioneer, South Korea), 100 ng genomic DNA and 10 pmol of forward (CS-COX 4150F, 5′-AATCCAACATGYTTCTTCA-3′) and reverse primers (CS-COX 7780R, 5′-CAGGGATTTAACCTGCAATT-3′) (see also Song 2015). The amplification reaction was performed under the following program: an initial denaturation at 94 °C for 3 min, 35 cycles of a denaturation at 94 °C for 30 s, an annealing at 54 °C for 30 s and an elongation at 72 °C for 1 min, and a final elongation at 72 °C for 7 min. After confirming the success of amplification on 1.5% agarose gel, PCR products were purified using AccuPrep^®^ PCR purification kit (Bioneer). All purified PCR products were sent for commercial sequencing at Macrogen Inc. (Seoul, South Korea) using the ABI PRISM BigDye™ Terminators v3.1 Cycle Sequencing Ready Reaction Kit (Applied Biosystems, USA), the ABI 3730xl DNA analyzer (Applied Biosystems) and the primers used in the PCR reaction. The Chromatogram for each sequence was checked using Sequencher 4.1.4 (Gene Codes Corporation). No deletions or insertions were found from all obtained COI sequences consisting of 1551 bp.

### Phylogeographic analysis

Haplotypes were identified using DnaSP 6.12.03 (Rozas et al. 2017). Population-level genetic diversity indices, such as haplotype diversity (*H*_d_), nucleotide diversity (π), number of haplotypes (*h*) and pairwise *F*_ST_ were estimated using DnaSP. A Median-joining haplotype network was constructed using PopART (Leigh and Bryant 2015) under its default settings, and the output network was edited in Affinity Designer software after being exported as a vector image.

We used BEAST 2.6.3 (Bouckaert et al. 2019) to estimate the phylogenetic relationship and divergence times among haplotypes. Input files were generated using BEAUTI, and the dataset was designed to include all haplotypes of *Coreoleuciscus* species as well as the haplotypes from the genera *Pseudorasbora* (from China, Korea), *Pungtungia* (from Japan) *Pseudopungtungia* (from Korea) and *Gnathopogon* (from Korea and China) that were used as outgroups. The outgroup species were selected based on both the phylogenetic relationships and the geographic proximity. We used a relaxed uncorrelated lognormal clock model for the variation of substitution rates along each branch and a Yule prior for the relative node times in the tree. The best-fit substitution model was determined using jModelTest 2.1 (Darriba et al. 2012). The Posterior distribution of parameters was estimated using 10,000,000 MCMC generations sampled every 1000th generation. The convergence of the chains was checked using Tracer 1.7.1 (Rambaut et al. 2018). We used TreeAnnotator to discard the initial 20% as burn-in and to generate maximum clade credibility of a consensus tree. Since no fossil record has been reported for *Coreoleuciscus*, we applied the fossil calibration points of two species that are known as the closest taxon to *Coreoleuciscus* based on previous phylogenetic studies: 5.3 MYA for a *Gnathopogon macrocephala* fossil from Late Miocene (Zhou 1990), and 3.0 MYA for the oldest *Pseudorasbora*-like fossil record (Young and Tchang 1936).

To infer the ancestral geographic range of the *Coreoleuciscus* populations, we conducted a Bayesian Binary MCMC (BBM) analysis that was implemented in RASP (Yu et al. 2015). BBM analysis is a particularly robust tool for taxa with evident discontinuities in distribution, such as freshwater fish species with distributions restricted by stream structures (Zhao et al. 2013). Our fossil-calibrated chronogram from BEAST was applied to the BBM analysis. We defined six target geographic areas for *Coreoleuciscus;* Han + Eastern, Geum + Ungcheon, Mangyeong, Seomjin, Nakdong and Samhwa, and two outgroup areas, Japan and China where the outgroup species (*Gnathopogon*, *Pseudorasbora*, Pungtungia) are distributed. We then assigned the maximum number of areas for each node to be 4. The analysis was run under the default setting for other parameters.

## Result

### Genetic diversity

A total of 73 complete COI haplotypes (1551 bp) were obtained from 31 *Coreoleuciscus* populations (Table 2). The mean number of haplotypes and haplotype diversity were higher in *C. splendidus* (mean *h* = 3.77, mean *H*_d_ = 0.34) than *C. aeruginos* (mean *h* = 2.61, mean *H*_d_ = 0.22) (Table 2). *C. splendidus* populations in the Han River exhibited the highest level of diversity (mean *h* = 5.75, mean *H*_d_ = 0.52) (Table 2). The highest genetic diversity in *C. aeruginos* was observed from the Nakdong River populations (mean *h* = 3.14, mean *H*_d_ = 0.27), which was lower than that of the Han River populations in *C. splendidus*, though the Nakdong River is a fairly large river similar in size to the Han River. Populations from small-sized drainages, including Ungcheon, Mangyeong, Samhwa and Yeongdeokosip, have lower haplotype diversity (mean *h* = 1.42, mean *H*_d_ = 0.12) than the populations from major river basins (Han, Geum, Seomjin and Nakdong; mean *h* = 3.8, mean *H*_d_ = 0.33). Even in the same species, there were few haplotypes shared between drainages with high levels of pairwise-*F*_ST_ values (mean *F*_ST_ between Han and Geum = 0.83: mean *F*_ST_ between Nakdong and Seomjin = 0.95) (Table S1).

**Table 2:** Genetic diversity of each *Coreoleuciscus* population that is represented by population codes used in the Table 1

Population	# of haplotype (*h*)	Haplotype diversity (*H*_d_)	Nucleotide diversity (π)
CS-H-IJ	3	0.27895	0.00030
CS-H-HT	5	0.77895	0.00092
CS-H-JJ	7	0.69474	0.00056
CS-H-HC	6	0.57368	0.00043
CS-H-PC	1	0.00000	0.00000
CS-H-HI	8	0.64052	0.00072
CS-H-SG	4	0.28421	0.00019
CS-H-DC	12	0.92105	0.00125
CS-H-SO	1	0.00000	0.00000
CS-H-GN	2	0.52500	0.00068
CS-H-YG	1	0.00000	0.00000
CS-G-SD	8	0.64737	0.00076
CS-G-YG	3	0.57310	0.00040
CS-G-YD	1	0.00000	0.00000
CS-G-GC	1	0.00000	0.00000
CS-G-GM	3	0.19474	0.00013
CS-U-UC	1	0.00000	0.00000
CS-M-SY	1	0.00000	0.00000
CA-S-JW	3	0.19474	0.00013
CA-S-SC	1	0.00000	0.00000
CA-S-HG	1	0.00000	0.00000
CA-S-GR	3	0.42632	0.00030
CA-S-SH	1	0.00000	0.00000
CA-N-BH	3	0.27895	0.00019
CA-N-HJ	3	0.19474	0.00013
CA-N-BB	2	0.10000	0.00006
CA-N-YG	5	0.36842	0.00026
CA-N-MY	4	0.64737	0.00053
CA-N-GH	3	0.19474	0.00013
CA-N-DC	2	0.10000	0.00013
CA-N-YO	3	0.35870	0.00024

### Haplotype network

**Fig. 2:**
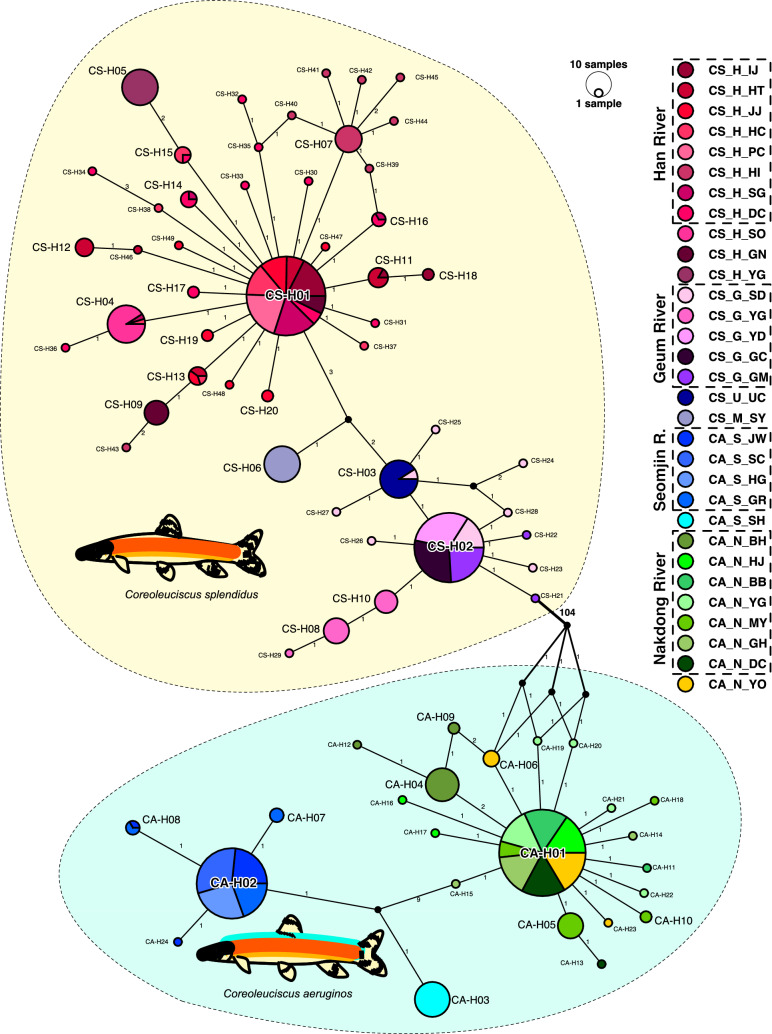
COI haplotype network of *Coreoleuciscus*. Each value along the line indicates the mutation steps. Yellow and blue shades surrounded with the dashed lines represent the haplogroups of *C. splendidus* and *C. aeruginos*, respectively. Circle size represents the number of individuals that shared an identical haplotype (color figure online)

Strong differentiation was found between the two *Coreoleuciscus* species in the haplotype network (Fig. 2). Most individuals were almost perfectly assigned to their population or drainage by COI haplotyping, though some individuals from Ungcheon, Yeongdeokosip, and other east coastal river populations, were exceptionally clustered with other drainage populations (Fig. 2). Star-like patterns in the network indicate that there may have been historically explosive expansions of the populations in the Nakdong and Han River. Two haplotypes, CS-H01 from the Han River and CA-H01 from the Nakdong River, appeared across the populations within the rivers without exception (Fig. 2).

### Divergence time and ancestral range estimation

**Fig. 3:**
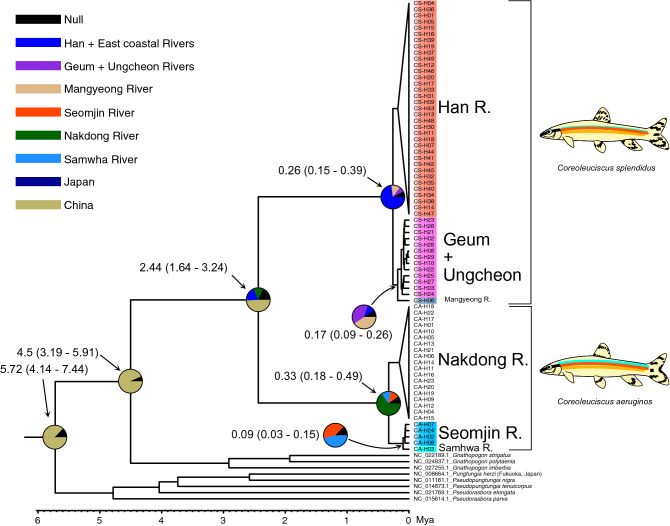
Divergence time and ancestral range of *Coreoleuciscus* species inferred by BEAST and RASP, respectively. Value on each node represents the mean divergence time (million years, Mya) with 95% CI. Pie graphs indicate the ancestral distribution as relative probabilities at the time of differentiation (around the node)

A strong differentiation was observed between the two *Coreoleuciscus* species in our BEAST tree (Fig. 3). The drainage-level monophyly was also well-supported with strong posterior probabilities (Fig. 3). The divergence between the two sister genera, *Coreoleuciscus* and *Gnathopogon*, was estimated to be 4.5 MYA [3.19–5.91 95% highest posterior density (HPD)] (Fig. 3). The time to most recent common ancestor (TMRCA) of all *Coreoleuciscus* populations was estimated to be 2.44 MYA (1.64–3.24 95% HPD), which corresponds to the Late Plio-Pleistocene (Fig. 3). The first inter-drainage split of *Coreoleuciscus* populations was estimated to occur around the Late Pleistocene (Fig. 3). Based on the results of our ancestral distribution reconstruction, MRCA of *Coreoleuciscus* was thought to have been distributed in eastern Eurasian continent (Fig. 3; Table 3). Two *Coreoleuciscus* species were subsequently dispersed into WKS (Han River) and SKS (Nakdong River) of the Korean Peninsula, respectively (Fig. 3). Perhaps, after the first colonization, *C. splendidus* was further dispersed to the south and *C. aeruginos* to the west (Fig. 3).

## Discussion

### Colonization history of ancestral populations

In this study, mitochondrial COI haplotypes were analyzed from 31 *Coreoleuciscus* populations to estimate the distribution of genetic diversity and infer the phylogeographic history on the Korean Peninsula. A number of previous studies have suggested that Korean endemic freshwater fishes are mainly products of vicariance by the orogenic activities during the Late Cretaceous-Paleocene (Kim and Bang 2012; Kim et al. 2012; Kwan et al. 2017). In contrast, our results show that the both ancestral populations colonized the southwestern part of the Korean Peninsula when the drainage systems were forming confluence during the Late Plio-Pleistocene, which postdates the period of the orogenic events having divided the subdistricts (Kim et al. 2012). Considering the mismatch between divergence time of *Coreoleuciscus* and the presence of the BMR, it is more reasonable to view the products of allopatric speciation caused by the independent dispersal and colonization at different locations of the two ancestral *Coreoleuciscus* populations, rather than the geographical severance itself caused by tectonic events led to the speciation of the two species. Our results are in line with other studies having shown that various freshwater species of the Korean Peninsula originated from continental regions during the Pleistocene (Tominaga et al. 2016; Jeon 2018; Jang-Liaw et al. 2019; Won et al. 2020). In conclusion, the phylogenetic background of the rheophilic *Coreoleuciscus* species can be explained by the colonizer hypothesis.

**Table 3:** Estimated divergence time and ancestral distributional area on each node

Node	Estimated age (MYA)		Ancestral area
	Mean	95% CI	
Samhwa River/Seomjin River	0.09	0.03–0.16	Samhwa River
Seomjin + Samhwa River/Nakdong River	0.33	0.18–0.49	Nakdong River
Crown Nakdong River	0.16	0.08–0.24	Nakdong River
Crown Seomjin River	0.06	0.01–0.10	Seomjin River
Crown Han River	0.18	0.11–0.26	Han River
Han River/Geum + Ungcheon + Mangyeong River	0.26	0.15–0.39	Han River
Geum + Ungcheon/Mangyeong River	0.17	0.09–0.26	Mangyeong River
Crown Ungcheon + Geum River	0.12	0.06–0.19	Geum River
*C. splendidus*/*C. aeruginos*	2.44	1.64–3.24	China
Coreoleuciscus/Gnathopogon	4.50	3.19–5.91	China
*Coreoleuciscus* + *Gnathopogon*/*Pseudorasbora* + *Pungtungia* sensu lato	5.72	4.14–7.44	China

### Interpopulation divergence

At the population level, it was shown in our results that *Coreoleuciscus* is largely divided into four major lineages, Han vs. non-Han [Geum + Ungcheon + Mangyeong River] in *C. splendidus*, and Nakdong vs. non-Nakdong in *C. aeruginos*, which has also been suggested in a previous study (Song et al. 2010). Based on our results, two ancestral *Coreoleuciscus* populations may have begun to disperse from the Eurasian continent to westward when the sea levels were lower than at present during the Quaternary, forming the contemporary distribution structure by inter-drainage dispersal events during the Pleistocene. Both Han and non-Han populations in *C. splendidus* were originally distributed in the paleo-Han River, while some populations or individuals have perhaps had the opportunity to migrate and colonize the freshwater systems at the southern part of the peninsula. The fact that there are many shared haplotypes among non-Han drainage populations supports this hypothesis. Our evidence support the hypothesis that there were the historical connectivities among paleo-rivers on the southwestern part of the Korean Peninsula, which may also explain the historical reasons for the distributional range of some Korean endemic species such as *Pseudopuntungia nigra*, *Liobagrus obesus* (Kim 1997), which are currently distributed in Geum and Mangyeong River.

Considering the history of colonization, the path of *C. aeruginos* or its ancestor migrating to the Nakdong River, which is quite south from the WKS, is rather questionable. There are two possible scenarios can be suggested to explain such a long-distance colonization of ancestral *C. aeruginos* population(s). First, there may have been connections between watersheds of the Han and Nakdong River due to ‘river capture’ associated with geological fluctuations, albeit temporarily, which may have resulted in the formation of a population *C. aeruginos* in a drainage flowing to the other coastal side. This scenario can be supported by cases with similar distributional characteristics to *Coreoleuciscus*. For example, *Koreocobitis naktongensis*, an endemic species to the Nakdong River is a very close sister species of *K. rotundicaudata*, an species endemic to the Han River. Second, it is worth considering ‘estuary coalescence’, though the distance between the estuaries of the two rivers are quite large compared to the distance between the watersheds. Under this scenario, there was a huge confluence extending from the paleo-Yellow water system with the sea level fluctuation during the Pleistocene, through which the individuals (ancestral *C. aeruginos*) would have serially extended their distributions to the east of the Korean Peninsula.

### Historical and artificial colonization in east coastal system

Our results revealed that the east coastal populations of *C. splendidus* had a strong genetic affinity with the Han River populations. Even for this reason, these east coastal populations of *C. splendidus* have been considered as an artificially introduced populations from the Han River (Byeon and Oh 2015). However, in the case of the Samcheokosip population, it has been known for a long time that there has been river capture with the Han River, and historically this area has been considered to have a natural population (Choi 1973; Kim et al. 2017). In a previous phylogeographic study, it could be estimated that these two populations (Samcheokosip stream and Han River) had gene flow during the Late Pleistocene, probably via river capture, as a result of analysis using mitochondrial cytochrome *b* sequence data (Kim et al. 2017). Since our data showed that the Samcheokosip stream population shared haplotypes with some populations in the Han River, however, the possibility that there may have been more recent gene flow between these two regions should be considered. It is still too early to draw a conclusion on this, and further data analysis is needed.

Since the inhabitation of *C. splendidus* in Gangneungnamdae and Yeongok streams was first reported recently (Byeon and Oh 2015), we expected that these populations were likely to share the haplotypes found in the Han River, assuming anthropogenic introduction considering the fact that a substitution rate in mitochondria reflect a historical divergence. However, Yeongok stream population consisted of a single unique haplotype, CS-H05. On the other hand, Gangneungnamdae stream population contained CS-H01, a haplotype commonly found in the Han River along with CS-H05. Given our extensive sampling coverage throughout the Han River, this result indicates that the Yeongok stream population might have diverged historically. On the other hand, the composition of both shared and unique haplotypes in the Gangneungnamdae population is presumed to be a mixture between introduced and historically diverged lineage though a more detailed investigation is required to draw conclusions on it.

Since the discovery of *C. aeruginos* in the Yeongdeokosip stream was relatively recent (Song 2015), the population can be considered as introduced, though it is not yet clear where this population originated. Judging from our results, it is highly likely that it originated from the Nakdong River. Given that it was recently discovered, it might artificially be displaced. Recent research results give hints on how it was introduced. *Squalidus gracilis majimae* inhabits only rivers flowing to the west and south coasts of the Korean Peninsula, and strangely, traces of introgression into the gene pool of *S. multimaculatus* originally inhabiting these rivers have been recently discovered (Lee et al. 2017; Jeon et al. 2018). Therefore, it can be strongly suspected that stocks of fish communities in the Nakdong River were artificially transferred to this area.

### Conservation implications

*Coreoleuciscus* populations with a high level of genetic diversity were found to be distributed mainly along the BMR in this study. It can be estimated that mountain streams belonging to the major drainage basins of the Korean Peninsula and existing along the BMR served as glacial refugia for populations of *Coreoleuciscus* species (Aizawa et al. 2012; Chung et al. 2018; Jang et al. 2021). Since *Coreoleuciscus* species prefer to inhabit cold water areas with rapid current and high dissolved oxygen (Song and Bang 2015; Song et al. 2020), it can be presumed that their ancestors had historical routes from estuary reaching to the upstream areas. Considering this point, it seems clear that *Coreoleuciscus* populations began to form during the Late Plio-Pleistocene, when the drainage structure on the Korean Peninsula was completed (Dias et al. 2014; Zhang et al. 2019). Combining our biogeographic assumptions and hypotheses presented above, it is well explained that *Coreoleuciscus* species only inhabit the Korean Peninsula, the only region in East Asia with a uniquely developed mountainous topography.

In the haplotype network, haplotypes representing four major drainage basins existed, each of which a star-like distribution was observed. This can be taken to mean that the sudden expansion in population size was made for each drainage system, after the historical isolation between the water systems was completed. Among all drainage systems, the Han River populations exhibited the highest genetic diversity. The major haplotype in the Han River, CS-H01, was found with high frequency across most of the populations, indicating that the historical gene flow has been maintained active among the populations. There are not many studies on the gene flow between regions within the Han River. In a recent study on fluvial sculpin, *Cottus koreanus*, however, a distinct genetic structure was revealed in the Han River, indicating the signature of spatial isolation among the populations (Baek et al. 2018). In our study, an opposite result was obtained, which may be attributed to differences in behavioral radius and migration ability between *Cottus* and *Coreoleuciscus*.

The active gene flow among the populations in the water system may have been the driving force that maintained the genetic diversity of these populations. However, some populations consisted of only unique haplotypes. The Yugu stream of the Geum River (*C. splendidus*) and the Bonghwa (*C. aeruginos*) of the Nakdong River are the examples. It is possible that these populations have historically had very little interaction with other within-drainage populations for some reason. Since populations that have undergone fragmentation are more likely to have lost genetic diversity (Lynch et al. 1995; Fahrig 2003), special conservation management of these populations needs to be entailed.

There are several populations that are completely spatially isolated with very low genetic diversity. These small populations can be highly responsive to even minute environmental changes, which can lead to local extinction (Lynch et al. 1995). In *C. aeruginos*, the population of Namhae Island (Samhwa River) is likely to be very susceptible to anthropogenic disturbances because the population inhabit the spatially very small basin. Surprisingly, only three haplotypes were found in *C. aeruginos* of the Seomjin River, though it is a large drainage basin. The *C. splendidus* populations in the Mangyeong and Ungcheon rivers, which are small in size, also showed monomorphism in COI haplotypes. The populations of these streams require special management, and a systematic conservation plan should be established to avoid local extinction.

## Supplementary Information

Below is the link to the electronic supplementary material.

Supplementary file1 (DOCX 39 kb)

## Data Availability

All mtDNA COI haplotypes were deposited in GenBank (accession numbers from MW652418to MW652466 for *C. splendidus* and from MW652516 to MW652539 for *C. aeruginos*).
