# Supplementary material for: Phylogeography of the Korean endemic Coreoleuciscus (Cypriniformes: Gobionidae): the genetic evidence of colonization through Eurasian continent to the Korean Peninsula during Late Plio-Pleistocene
Source: Genes Genomics. 2022 Apr 19;44(6):709–19. doi: 10.1007/s13258-022-01243-y (PMC9120112; doi:10.1007/s13258-022-01243-y)
Supplement: Supplementary file 1 — Supplementary file1 (DOCX 39 kb) [file 13258_2022_1243_MOESM1_ESM.docx]

Supplementary Table S1. Pairwise *F*_ST_ matrix among *Coreoleuciscus* populations. CS and CA represent *C. splendidus* and *C. aeruginos*, respectively.

|  |  | CS | | | | | | | | | | | | | | | | | | CA | | | | | | | | | | | |
| --- | --- | --- | --- | --- | --- | --- | --- | --- | --- | --- | --- | --- | --- | --- | --- | --- | --- | --- | --- | --- | --- | --- | --- | --- | --- | --- | --- | --- | --- | --- | --- |
|  |  | H-HT | H-IJ | H-HC | H-JJ | H-PC | H-DC | H-SG | H-HI | H-YG | H-GN | H-SO | G-SD | G-YG | G-GM | G-GC | G-YD | U-UC | M-SY | S-JW | S-GR | S-HG | S-SC | S-SH | N-HJ | N-BH | N-YG | N-MY | N-DC | N-GH | N-YO |
| CS | H-IJ | 0.13 |  |  |  |  |  |  |  |  |  |  |  |  |  |  |  |  |  |  |  |  |  |  |  |  |  |  |  |  |  |
|  | H-HC | 0.16 | 0.06 |  |  |  |  |  |  |  |  |  |  |  |  |  |  |  |  |  |  |  |  |  |  |  |  |  |  |  |  |
|  | H-JJ | 0.14 | 0.05 | 0.03 |  |  |  |  |  |  |  |  |  |  |  |  |  |  |  |  |  |  |  |  |  |  |  |  |  |  |  |
|  | H-PC | 0.21 | 0.08 | 0.05 | 0.04 |  |  |  |  |  |  |  |  |  |  |  |  |  |  |  |  |  |  |  |  |  |  |  |  |  |  |
|  | H-DC | 0.12 | 0.04 | 0.02 | 0.03 | 0.03 |  |  |  |  |  |  |  |  |  |  |  |  |  |  |  |  |  |  |  |  |  |  |  |  |  |
|  | H-SG | 0.18 | 0.05 | 0.02 | 0.03 | 0.00 | 0.00 |  |  |  |  |  |  |  |  |  |  |  |  |  |  |  |  |  |  |  |  |  |  |  |  |
|  | H-HI | 0.48 | 0.57 | 0.53 | 0.50 | 0.64 | 0.40 | 0.58 |  |  |  |  |  |  |  |  |  |  |  |  |  |  |  |  |  |  |  |  |  |  |  |
|  | H-YG | 0.79 | 0.93 | 0.89 | 0.87 | 1.00 | 0.75 | 0.95 | 0.88 |  |  |  |  |  |  |  |  |  |  |  |  |  |  |  |  |  |  |  |  |  |  |
|  | H-GN | 0.35 | 0.45 | 0.39 | 0.34 | 0.53 | 0.30 | 0.47 | 0.58 | 0.87 |  |  |  |  |  |  |  |  |  |  |  |  |  |  |  |  |  |  |  |  |  |
|  | H-SO | 0.63 | 0.82 | 0.73 | 0.70 | 1.00 | 0.49 | 0.86 | 0.78 | 1.00 | 0.75 |  |  |  |  |  |  |  |  |  |  |  |  |  |  |  |  |  |  |  |  |
|  | G-SD | 0.82 | 0.87 | 0.86 | 0.84 | 0.90 | 0.78 | 0.88 | 0.85 | 0.94 | 0.85 | 0.92 |  |  |  |  |  |  |  |  |  |  |  |  |  |  |  |  |  |  |  |
|  | G-YG | 0.88 | 0.93 | 0.92 | 0.91 | 0.96 | 0.85 | 0.94 | 0.91 | 0.97 | 0.90 | 0.96 | 0.60 |  |  |  |  |  |  |  |  |  |  |  |  |  |  |  |  |  |  |
|  | G-GM | 0.88 | 0.95 | 0.93 | 0.92 | 0.98 | 0.85 | 0.96 | 0.91 | 0.99 | 0.91 | 0.99 | 0.08 | 0.76 |  |  |  |  |  |  |  |  |  |  |  |  |  |  |  |  |  |
|  | G-GC | 0.90 | 0.96 | 0.95 | 0.93 | 1.00 | 0.86 | 0.98 | 0.93 | 1.00 | 0.93 | 1.00 | 0.09 | 0.81 | 0.00 |  |  |  |  |  |  |  |  |  |  |  |  |  |  |  |  |
|  | G-YD | 0.90 | 0.96 | 0.95 | 0.93 | 1.00 | 0.86 | 0.98 | 0.93 | 1.00 | 0.93 | 1.00 | 0.09 | 0.81 | 0.00 | 0.00 |  |  |  |  |  |  |  |  |  |  |  |  |  |  |  |
|  | U-UC | 0.88 | 0.96 | 0.94 | 0.92 | 1.00 | 0.84 | 0.97 | 0.92 | 1.00 | 0.91 | 1.00 | 0.49 | 0.88 | 0.91 | 1.00 | 1.00 |  |  |  |  |  |  |  |  |  |  |  |  |  |  |
|  | M-SY | 0.85 | 0.95 | 0.92 | 0.90 | 1.00 | 0.80 | 0.96 | 0.90 | 1.00 | 0.90 | 1.00 | 0.86 | 0.94 | 0.98 | 1.00 | 1.00 | 1.00 |  |  |  |  |  |  |  |  |  |  |  |  |  |
| CA | S-JW | 0.99 | 1.00 | 1.00 | 1.00 | 1.00 | 0.99 | 1.00 | 0.99 | 1.00 | 0.99 | 1.00 | 0.99 | 1.00 | 1.00 | 1.00 | 1.00 | 1.00 | 1.00 |  |  |  |  |  |  |  |  |  |  |  |  |
|  | S-GR | 0.99 | 1.00 | 0.99 | 0.99 | 1.00 | 0.99 | 1.00 | 0.99 | 1.00 | 0.99 | 1.00 | 0.99 | 1.00 | 1.00 | 1.00 | 1.00 | 1.00 | 1.00 | 0.03 |  |  |  |  |  |  |  |  |  |  |  |
|  | S-HG | 0.99 | 1.00 | 1.00 | 1.00 | 1.00 | 0.99 | 1.00 | 1.00 | 1.00 | 1.00 | 1.00 | 0.99 | 1.00 | 1.00 | 1.00 | 1.00 | 1.00 | 1.00 | 0.00 | 0.08 |  |  |  |  |  |  |  |  |  |  |
|  | S-SC | 0.99 | 1.00 | 1.00 | 1.00 | 1.00 | 0.99 | 1.00 | 1.00 | 1.00 | 1.00 | 1.00 | 0.99 | 1.00 | 1.00 | 1.00 | 1.00 | 1.00 | 1.00 | 0.00 | 0.08 | 0.00 |  |  |  |  |  |  |  |  |  |
|  | S-SH | 0.99 | 1.00 | 1.00 | 1.00 | 1.00 | 0.99 | 1.00 | 1.00 | 1.00 | 1.00 | 1.00 | 0.99 | 1.00 | 1.00 | 1.00 | 1.00 | 1.00 | 1.00 | 0.95 | 0.90 | 1.00 | 1.00 |  |  |  |  |  |  |  |  |
|  | N-HJ | 0.99 | 1.00 | 1.00 | 1.00 | 1.00 | 0.99 | 1.00 | 0.99 | 1.00 | 0.99 | 1.00 | 0.99 | 1.00 | 1.00 | 1.00 | 1.00 | 1.00 | 1.00 | 0.98 | 0.97 | 0.99 | 0.99 | 0.99 |  |  |  |  |  |  |  |
|  | N-BH | 0.99 | 1.00 | 1.00 | 0.99 | 1.00 | 0.99 | 1.00 | 0.99 | 1.00 | 0.99 | 1.00 | 0.99 | 1.00 | 1.00 | 1.00 | 1.00 | 1.00 | 1.00 | 0.98 | 0.97 | 0.99 | 0.99 | 0.99 | 0.89 |  |  |  |  |  |  |
|  | N-YG | 0.99 | 1.00 | 1.00 | 0.99 | 1.00 | 0.99 | 1.00 | 0.99 | 1.00 | 0.99 | 1.00 | 0.99 | 1.00 | 1.00 | 1.00 | 1.00 | 1.00 | 1.00 | 0.97 | 0.96 | 0.98 | 0.98 | 0.98 | 0.00 | 0.85 |  |  |  |  |  |
|  | N-MY | 0.99 | 0.99 | 0.99 | 0.99 | 1.00 | 0.99 | 0.99 | 0.99 | 1.00 | 0.99 | 1.00 | 0.99 | 0.99 | 1.00 | 1.00 | 1.00 | 1.00 | 1.00 | 0.96 | 0.95 | 0.96 | 0.96 | 0.96 | 0.32 | 0.80 | 0.28 |  |  |  |  |
|  | N-DC | 0.99 | 1.00 | 1.00 | 1.00 | 1.00 | 0.99 | 1.00 | 0.99 | 1.00 | 0.99 | 1.00 | 0.99 | 1.00 | 1.00 | 1.00 | 1.00 | 1.00 | 1.00 | 0.98 | 0.97 | 0.99 | 0.99 | 0.99 | 0.00 | 0.89 | 0.00 | 0.27 |  |  |  |
|  | N-GH | 0.99 | 1.00 | 1.00 | 1.00 | 1.00 | 0.99 | 1.00 | 0.99 | 1.00 | 0.99 | 1.00 | 0.99 | 1.00 | 1.00 | 1.00 | 1.00 | 1.00 | 1.00 | 0.98 | 0.97 | 0.99 | 0.99 | 0.99 | 0.00 | 0.89 | 0.00 | 0.32 | 0.00 |  |  |
|  | N-YO | 0.99 | 1.00 | 1.00 | 0.99 | 1.00 | 0.99 | 1.00 | 0.99 | 1.00 | 0.99 | 1.00 | 0.99 | 1.00 | 1.00 | 1.00 | 1.00 | 1.00 | 1.00 | 0.97 | 0.96 | 0.98 | 0.98 | 0.98 | 0.07 | 0.86 | 0.05 | 0.31 | 0.07 | 0.07 |  |
|  | N-BB | 0.99 | 1.00 | 1.00 | 1.00 | 1.00 | 0.99 | 1.00 | 0.99 | 1.00 | 0.99 | 1.00 | 0.99 | 1.00 | 1.00 | 1.00 | 1.00 | 1.00 | 1.00 | 0.99 | 0.98 | 1.00 | 1.00 | 1.00 | 0.00 | 0.91 | 0.00 | 0.35 | 0.00 | 0.00 | 0.08 |
